# The Genome Database for Rosaceae (GDR): year 10 update

**DOI:** 10.1093/nar/gkt1012

**Published:** 2013-11-12

**Authors:** Sook Jung, Stephen P. Ficklin, Taein Lee, Chun-Huai Cheng, Anna Blenda, Ping Zheng, Jing Yu, Aureliano Bombarely, Ilhyung Cho, Sushan Ru, Kate Evans, Cameron Peace, Albert G. Abbott, Lukas A. Mueller, Mercy A. Olmstead, Dorrie Main

**Affiliations:** ^1^Department of Horticulture, Washington State University, Pullman, WA 99164-6414, USA, ^2^Department of Genetics and Biochemistry, Clemson University, Clemson, SC 29634, USA, ^3^Boyce Thompson Institute for Plant Research, Tower Road, Ithaca, NY 14853, USA, ^4^Department of Computer Science, Saginaw Valley State University, University Center, MI 48710, USA and ^5^Horticultural Sciences Department, University of Florida, Gainesville, FL 32611, USA

## Abstract

The Genome Database for Rosaceae (GDR, http:/www.rosaceae.org), the long-standing central repository and data mining resource for Rosaceae research, has been enhanced with new genomic, genetic and breeding data, and improved functionality. Whole genome sequences of apple, peach and strawberry are available to browse or download with a range of annotations, including gene model predictions, aligned transcripts, repetitive elements, polymorphisms, mapped genetic markers, mapped NCBI Rosaceae genes, gene homologs and association of InterPro protein domains, GO terms and Kyoto Encyclopedia of Genes and Genomes pathway terms. Annotated sequences can be queried using search interfaces and visualized using GBrowse. New expressed sequence tag unigene sets are available for major genera, and Pathway data are available through FragariaCyc, AppleCyc and PeachCyc databases. Synteny among the three sequenced genomes can be viewed using GBrowse_Syn. New markers, genetic maps and extensively curated qualitative/Mendelian and quantitative trait loci are available. Phenotype and genotype data from breeding projects and genetic diversity projects are also included. Improved search pages are available for marker, trait locus, genetic diversity and publication data. New search tools for breeders enable selection comparison and assistance with breeding decision making.

## INTRODUCTION

The Genome Database for Rosaceae (GDR) is a comprehensive online database resource of curated genomic, genetic and breeding data and analysis tools for the Rosaceae research community. The Rosaceae family includes many crops of economic and nutritional importance such as almond, apple, apricot, blackberry, cherry, peach, pear, plum, raspberry, rose and strawberry. In 2012, the value of production of Rosaceae crops exceeded $12.56 billion, representing 48% of all the fruit and nut crops produced in the USA (1). Composed of species with a wide variety of form, habit, function and ploidy, the Rosaceae family is considered an interesting biological system for studying fundamental questions in plant biology (2). In the past few years, whole-scale improvements in biological and computational technologies have enabled researchers to generate a growing wealth of genomic, genetic and breeding data for organisms of interest. For GDR, these new data include sequencing/re-sequencing several Rosaceae species, and identifying quantitative trait loci (QTL), Mendelian trait loci (MTL), genes and markers for important agricultural traits from large-scale genotype/phenotype/transcriptome datasets.

The GDR was established in 2003 to integrate publicly available genetic and genomic data and to provide genome analysis tools for the worldwide Rosaceae genomics research community (3). Initially, genomic data consisted mostly of expressed sequence tags (ESTs), which were collected, assembled into genus-specific unigene sets and functionally annotated, where possible, by association with known protein homologs. Genetic data such as linkage maps and genetic markers were also collected and integrated in the database. The GDR’s web interface and analysis tools were developed to enable data sharing and further sequence analysis (3).

In the past 5 years, the GDR has markedly expanded to accommodate new data types and an increase in data volume (Table 1), with significant infrastructure development and curation efforts. In addition, the GDR has been rebuilt using Tripal, a toolkit for construction of online biological databases (4,5). Tripal uses the generic, modular, ontology-driven and open-source database schema called Chado (6). In addition to storage of genomic and genetic data, Chado also enables storage of large-scale phenotypic and genotypic data using the recently added Natural Diversity tables (7). Tripal uses Drupal (http://drupal.org), a popular Content Management System for simplified creation of Web sites. Drupal enables non-programmers to contribute content, and simplifies development for programmers.

**Table 1.** Comparison of number of GDR entries between 2008 (previous publication in NAR) and 2013 by data type

Data type	Number of entries by year		Details
	2008	2013	
Genome	0	5	*P. persica* genome v1.0, *Malus* × *domestica* genome v1.0 and v1.0p, *F. vesca* genome v1.0 and v1.1
Gene	0	236 191	27 864 from the *P. persica* genome v1.0, 63 541 from the *Malus × domestica* genome v1.0, 30 294 from *Malus × domestica* genome v1.0p and 99 613 from *F. vesca* v1.0 and 14 879 from NCBI
Unigene	90 337	200 467	Unigene V5 from 503 851 NCBI ESTs (increased from Unigene V3 from 359 001 ESTs)
Marker	1700	2 229 311	Including 2 222 300 SNPs, 2623 AFLPs, 51 CAPs, 2468 SSRs, 30 Isozymes, 38 ISSRs, 496 RAPDs, 736 RFLPs, 23 STSs and 14 SCARs.
Genetic Map	37	84	10 371 loci including 8895 marker loci, 229 bins, 52 MTL and 1195 QTL
QTL	0	1195	QTL and MTL that are associated with 199 agronomic traits
MTLs	27	52	
Species	<100	516	Data available for 516 species, specific species pages with hyperlinks to various data and tools for 13 major species
Germplasm	0	8613	1814 cultivars, 5718 breeding research material, 1005 wild/unimproved and 76 population
Phenotype data	0	578 568	Data from 578 568 measurements using 481 trait descriptors for five crops (apple, peach, strawberry, tart cherry and sweet cherry)
Genotype data	0	28 296	Data from 28 296 measurements using 880 markers for 21 species
Publication	2447	5182	5182 publications from PubMED, USDA National Agricultural Library and conferences

## DATABASE DESCRIPTION

### GDR data and web interface

The GDR houses new genomic data such as whole-genome sequences with annotations of apple, peach and strawberry, the Rosaceae gene sequences from NCBI (8) anchored to the genome sequences and new EST unigene sets for major genera of Rosaceae (Table 1). Additionally, QTL data and more genetic maps and markers are now available. Pathways can be accessed through the new GDR Cyc Pathways databases, and synteny among the three sequenced genomes can be viewed through GBrowse_Syn (9). Collated genetic diversity data and publicly available breeding data are integrated with other relevant data.

### Genomics data

#### Whole genome sequence data

Currently available in the GDR are the whole genome assemblies: *Prunus persica* genome v1.0 (10), *Malus* × *domestica* genome v1.0 (11), *Malus* × *domestica* genome v1.0p, *Fragaria vesca* genome v1.0 (12) and *F. vesca* genome v1.1. The *Malus* × *domestica* genome v1.0 is represented as metacontigs, composed of assembled overlapping contigs that have been produced by the assembly of heterozygous genotypes and anchored with previously mapped genetic markers to the 17 chromosomes of apple (11). The v1.0p is the primary pseudo-haplotype assembly composed of chromosome-anchored contigs that are non-overlapping. Additional annotation of these assemblies by the GDR team included computational annotation of the predicted genes with homology to genes of closely related or plant model species and assignment of InterPro protein domains (13), GO terms and Kyoto Encyclopedia of Genes and Genomes database (KEGG) pathway and ortholog terms (14). Site visitors can access the assembly with annotations and the GDR functional annotations from respective genome pages, gene search pages and the graphical viewer, GBrowse (15,16).

The respective genome pages in GDR contain downloadable files, including Generic File Format (GFF) and FASTA formats, for the assembly with annotated gene predictions, homology, markers, single-nucleotide polymorphisms (SNPs) and repeats. Additionally, lists of annotated functional terms and Microsoft Excel files of protein homologs mapped via BLASTX (17) are available for downloading and contain hyperlinks to external databases as well as to GDR pages including GBrowse and gene or marker detail pages when applicable. The large USDA NIFA-funded SCRI project RosBREED (18) has provided re-sequencing data, including raw Illumina reads of multiple individuals from *Prunus* and *Malus* species available in FASTA format. Alignments of reads to respective genome assemblies are available in BAM format for *P. persica* v1.0 and *Malus × domestica* v1.0 genomes and can be accessed via respective genome pages. Genome alignments of the re-sequences are easily viewed without downloading of large BAM files by using a viewer such as the Integrative Genomics Viewer (19). From the *F. vesca* v1.0 and v1.1 genome pages, additional files are available for the chloroplast sequence and annotations, *Fragaria* transcript alignments, comparative alignments and gene index alignments.

**Figure 1.**
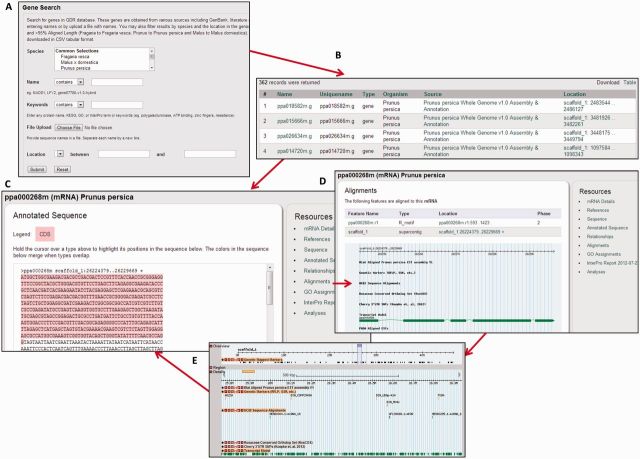
Gene search site in the GDR. (**A**) Genes can be searched by various categories, such as by species, name, genome location and keywords from functional annotation such as GO terms, InterPro protein domain name and KEGG pathway terms. A file with multiple names can be uploaded for searching as well. (**B**) The search results page has links for download, gene details page and GBrowse. (**C**) The gene details page has various tabs to show the data. The annotated sequence page is shown. (**D**) The alignment tab of the gene detail page, which shows the position in the whole genome and link to GBrowse. (**E**) A GBrowse page linked from the alignment tab of the gene detail page. Scientists can return to the gene detail page from GBrowse.

For researchers seeking specific sequences and genes for the above assemblies, the GDR provides a search site containing several query interfaces. Using these interfaces, users can conduct searches by gene name, genome location and association with computationally inferred functionality such as GO terms, InterPro domains and KEGG pathway terms (Figure 1A). This gene search site allows users to perform a query such as ‘Return all genes annotated with the word “resistance” between 1 Mb and 3.5 Mb on scaffold 1 of the peach genome’. Using the search site, site users can download the results or proceed to the gene details page within the GDR (Figure 1B).

The gene details page has several links in the resources sidebar to display the sequence and its motif annotations, genome alignments and homologies to sequences of other species in the GDR and other databases (Figure 1C). The alignment details provide links to view a gene in GBrowse (Figure 1D).

Using GBrowse, users can view the genomic features aligned to the genome, such as gene models, repeats, SNPs, alignments of ESTs, repeats, genetic markers and genes from other model plant species. Each feature in GBrowse is hyperlinked to a page with sequences and additional information, with hyperlinks to external databases where applicable.

The predicted genes from the whole genome sequences were used in the construction of PlantCyc (metabolic pathway) databases (20) using PathwayTools (21). Currently, three PlantCyc databases, PeachCyc, AppleCyc and FragariaCyc, are available in GDR. Gene models for peach, apple and strawberry were functionally annotated through a sequence homology search using the BLASTX algorithm (17) with the UniProtKB/Swiss-Prot (22) and TAIR Arabidopsis database (23). BLASTX matches with an expectation value <1e-20 were parsed, and EC codes were transferred to the Rosaceae gene models with Pathologic format (used by PathwayTools) using a Perl script, generate_pathologic_file.pl (https://github.com/solgenomics/sgn-home/blob/master/aure/scripts/solcyc/generate_pathologic_file.pl)

The orthologous regions among the three sequenced genomes of Rosaceae (24), as detected by the Mercator program (25), are displayed using a synteny browser GBrowse_Syn (9). GBrowse_Syn is hyperlinked to GBrowse for access to genomic annotations including markers from the conserved syntenic regions shown in GBrowse_Syn. Comparative genomics data made available in the GDR thus allows site visitors starting with one rosaceous genome to explore genomic features, anchored trait loci and genetic markers within orthologous regions of another rosaceous genome.

#### NCBI genes

Rosaceae sequences from the NCBI nucleotide database (8) were downloaded, parsed for gene, messenger RNA, CDS, 5'UTR and 3'UTR features and imported into the GDR. During parsing of the Rosaceae NCBI sequences, unique gene names were collected and a single non-redundant list of Rosaceae genes was combined to form a new GDR Gene Database. Multiple gene sequences, from different sources, are associated with their respective genes from the GDR Gene Database. The Rosaceae genes in the GDR Gene Database serve as a base entity for association with predicted genes from whole genome sequences, QTLs, genetic markers and mutant phenotypes as annotation progresses. Gene sequences from NCBI for all species of *Prunus*, *Malus* and *Fragaria* were mapped to the *P. persica* genome v1.0, *Malus × domestica* genome v1.0p and *F. vesca* genome v1.0, respectively. All NCBI-derived genes are searchable in the gene search page (Figure 1). Inclusion of the NCBI-derived genes, in addition to the predicted genes from whole genome assemblies, allows users to search genes by names used in publications (e.g. ‘*LFY1*’). The alignment of the NCBI-derived genes to the whole genome assembly will allow users to identify the predicted genes that match NCBI genes of their interest. Gene search using a name used in publications returns all the matching NCBI genes that are associated with the name and all the genomic locations where they are mapped physically. The gene search site also allows users to extract and download all the genes in a specified genomic region.

#### Rosaceae unigene and other transcript data

The GDR contains the publicly available Rosaceae ESTs downloaded from dbEST at NCBI. As reported by Jung *et al.* (3), unigene construction for the GDR occurs in four steps: (i) sequence filtering and trimming to obtain high-quality sequences, (ii) assembly into contigs to reduce the inherent redundancy, (iii) building unigene sets from the combined contigs and singlets and (iv) sequence annotation. A unigene v5.0 is available for each genus (*Prunus*, *Malus*, *Fragaria*, *Rosa* and *Pyrus*). Additionally, the assembled contigs and singlets for the five genera were combined to generate a unigene v5.0 for the entire Rosaceae. The *Prunus*, *Malus* and *Fragaria* unigenes are also mapped to the *P. persica* v1.0, *Malus × domestica* v1.0p and *F. vesca* v1.0 genomes, respectively. Other annotation for unigenes includes putative functional association by homology to genes in other species, InterPro protein domains and KEGG pathway terms. The GDR also houses annotated transcript data of strawberry submitted by collaborating scientists such as *Fragaria* 454 ESTs, complementary DNA from GenBank and mRNA from NCBI Reference Sequences (RefSeq, 26) that are aligned to the *F. vesca* genome v1.0.

Unigene pages, found under the species navigation menu, display details of the unigene assembly. The unigene pages have a resources sidebar with links to library information, homology, KEGG analysis, microsatellite analysis and downloads page. Unigenes can also be searched in the sequence search pages. Each unigene in the unigene s*et al.*o has a details page that provides contig information, and links to sequences, alignments, InterPro reports and homologs in various closely related or plant model species. The alignment of unigenes and other transcripts can be viewed in GBrowse and also downloaded from the respective genome pages.

### Genetic map, genetic diversity data, markers and trait loci

#### Genetic maps

GDR contains 84 genetic maps for Rosaceae species, including the reference maps for major crops: the TxE map (27,28) the reference map for *Prunus*, the FV × FN diploid *Fragaria* reference map (29) used in assembly of the *F. vesca* genome sequence and the rose-integrated consensus map built on information from four diploid populations and >1000 markers (30). Also available is the apple-integrated map that was developed for anchoring metacontigs from the whole-genome sequencing (11). The integrated map was derived from six F1 populations totaling 720 individuals.

CMap (31), the web-based graphical comparative map tool, can be used to view and compare maps from different cultivars and species. This comparative mapping facilitates information transfer from well-studied species to less-studied ones, especially in Rosaceae due to the essential collinearity between the genomes of *Prunus* and other genera of Rosaceae (2).

#### Genetic diversity data

The GDR contains DNA polymorphism data from various genetic diversity and breeding projects. Currently, data from nine diversity projects are available: three from *Prunus* and the remaining from *Malus* and *Pyrus* studies. The GDR also includes publicly available genotypic data of cultivars and breeding selections from breeding programs and projects, including RosBREED (18). Using the diversity search page, found under the search navigation menu, users can view the details of diversity projects and genotypes of the included germplasm and can filter results by study, dataset name, marker name, variety name or species. The search results a Microsoft Excel file, which includes marker name, allele, germplasm and dataset.

#### Genetic marker data

Details of the ∼2 million genetic markers used in genetic map development or genetic diversity studies, including SNPs, are available within the GDR. Marker annotations include marker aliases, source cultivar, source description, primer sequences, PCR conditions, literature references and map position where available. Genetic markers that have been anchored to the whole-genome sequences include SNP markers from array development projects such as the IRSC apple 9K (32), cherry 6K (33), UC Davis peach 6K (34) and IRSC peach 9K (35) arrays. These markers are also available to download in Microsoft Excel format from the genome pages and can be viewed in GBrowse.

**Figure 2.**
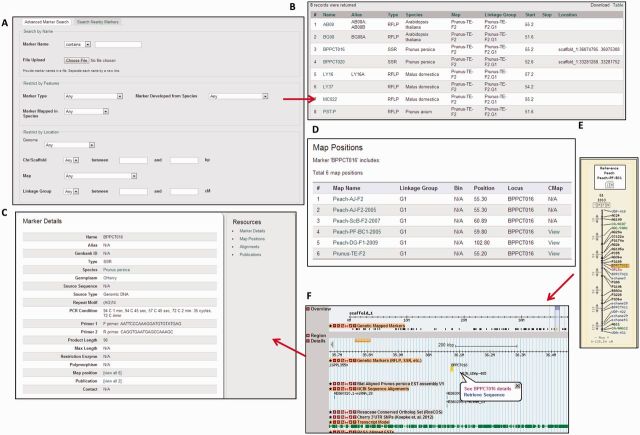
Marker search site in the GDR. (**A**) The advanced marker search site allows researchers to search markers by name, type, species and whole genome or genetic map locations. (**B**) The search results page shows information such as links to download data and the marker details page. (**C**) The marker details page with various tabs to show the detailed information. (**D**) The map position tab of the marker page shows all maps in which a marker has been mapped. (**E**) From the marker page, researchers can go to CMap. (**F**) For genome-anchored markers, CMap provides a hyperlink to GBrowse. From GBrowse, researchers can follow the link to go back to CMap, the marker details page or the Sequence Retrieval Tool.

Genetic markers can be accessed using the marker search page, which is located under the search menu item of the navigation menu. The search filter feature in the marker search page includes marker name, marker type, the species from which the marker is developed, the species to which the marker is mapped and map position in genetic map and genome (Figure 2). Additionally, users can upload a file of marker names for searching. Another search interface allows users to find markers near a targeted locus.

#### Trait locus data

Information about reported QTL/MTL is available in the GDR. This includes 1195 QTLs and 52 MTLs that are associated with 199 agronomic traits such as powdery mildew disease resistance, fruit skin color, ripening time and volatile organic compound content. QTLs are annotated in the GDR with aliases, curator-assigned QTL label, published symbol, curated trait category, trait name, taxon, trait description, screening method, map position, associated markers, statistical values and references. The search page for trait loci allows searching by trait locus type (QTL or MTL), species, curated trait category, trait name, published symbol and GDR-assigned label.

### Breeding data

**Figure 3.**
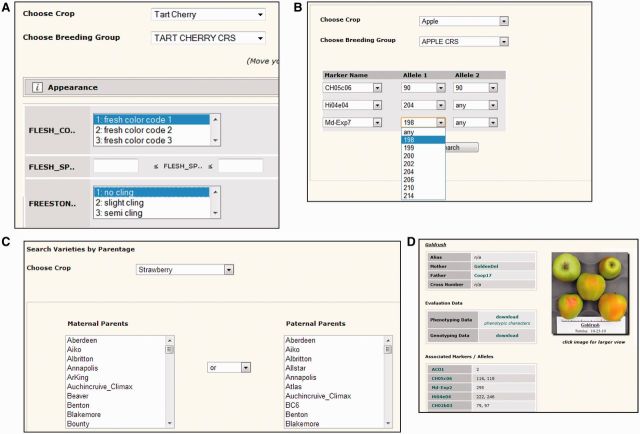
Search pages for breeding data in the GDR. (**A**) The search by phenotype page where researchers can search varieties by trait values, marker alleles (**B**) or parentage (**C**).

Breeding data stored in the GDR include phenotypic data, genotypic data, germplasm and pedigree data from the RosBREED project (www.rosbreed.org; 18) and the Washington Apple Breeding Program (36). Breeding data can be searched in various pages found under the Breeders Toolbox navigation menu. The search pages allow users to browse varieties by datasets and search for varieties with specific trait values, marker alleles and parentage (Figure 3). From the search results, users can view a list of varieties meeting specified criteria, download the results in a Microsoft Excel file or go to the variety details page.

### Publications

The GDR houses information about publications on Rosaceae genomics, genetics and breeding research. Details about publications were imported to the GDR from NCBI PubMed (http://www.ncbi.nlm.nih.gov/pubmed) and the USDA National Agricultural Library (NAL) (http://agricola.nal.usda.gov/databases/). Additionally, details of publications from other journals not present in these databases were added. Publications are updated in the GDR each week. Publications can be searched using a combination of keywords (in the abstract or title), all or partial titles, authors and other categories. Search results link to publication detail pages that contain the abstract, citation, external link to the full article and other details.

### Online tools

The GDR contains several online analysis tools found under the tools menu item on the navigation menu. New or enhanced analysis tools in the GDR include an instance of NCBI’s wwwBLAST tool (http://www.ncbi.nlm.nih.gov/staff/tao/URLAPI/wwwblast/), a custom Batch BLAST tool and a Sequence Retrieval Tool. The Batch BLAST server supports uploading of large datasets for pairwise comparison. It executes BLAST, and parses the output into a Microsoft Excel file. Scientists are notified by email when the job is complete and directed to a Web site to download result files. Currently, 54 GDR datasets are available in both BLAST servers for sequence comparison. The datasets include pseudomolecules, scaffolds, contigs, transcripts, CDS, peptides, proteins from the Peach v1.0, Apple v1.0 and Strawberry v1.1 and v1.0 genome sequences. Other datasets include NCBI dbESTs and GDR unigenes for various genera of Rosaceae, and Rosaceae Conserved Ortholog Set (37).

The sequence retrieval tool enables downloading of sequences including full chromosomes, scaffolds, genes, full transcripts, transcript coding sequences, proteins, genetic markers aligned to chromosomes, unigene contigs and ESTs. Users supply a list of sequence names to retrieve and can filter by a specific genome assembly, unigene or other project data. For features aligned to a whole genome, such as genes, transcripts and genetic markers, users can include a specified number of upstream and downstream bases in the retrieved sequence.

### Community resources

The GDR continues to provide community-based resources under the community navigation menu, including a US RosEXEC page, a RosIGI page, a conferences page, employment notices and mailing lists and message boards. The US RosEXEC (US Rosaceae Genomics, Genetics and Breeding Executive Committee) and RosIGI (Rosaceae International Genomic Initiative) serve as communication and coordination focal points for the research community. The US RosEXEC and RosIGI pages provide official documents, meeting minutes, membership and subcommittee information. Several mailing lists, in addition to the GDR mailing list, are available to serve the community with information for specific interests or purposes, and the archives can be viewed through the message board sites.

## CONCLUSION AND FUTURE DIRECTION

The addition and integration of annotated whole genome, trait locus and breeding data, and a substantial increase in genetic map and marker data, further enhance the GDR’s role as an essential resource for Rosaceae genomics, genetics and breeding research. The GDR enables use of genomic data toward the enhancement of Rosaceae crop performance and facilitates further understanding of the fundamental biology of rosaceous species. In addition, the reconstruction of the GDR using the open-source Tripal genome database toolkit allows the database to meet future emerging demands for storage of new data types and development of new web interfaces to accommodate scientists’ data-mining needs. With increases in the volume of data from high-throughput genotyping and re-sequencing projects of large numbers of germplasm, the GDR will be further enhanced to integrate the resulting large datasets. Future plans include associate traits and germplasm data with the developing Rosaceae Trait Ontology and development of web interfaces for registered users to upload various data, and curate gene models. A set of analysis tools for breeders is also under development to help breeders with operational decision making. Access to the GDR continues to grow annually. In 2012, the GDR had 40 222 visits by 14 237 unique visitors from 130 countries who accessed 176 259 pages.

## FUNDING

The USDA National Institute of Food and Agriculture–Specialty Crop Research initiative projects [2009-51181-06036, 2009-51181-05808]; Washington Tree Fruit Research Commission, Clemson University, University of Florida, Boyce Thompson Institute for Plant Research and Washington State University. Funding for open access charge: Federal grant.

*Conflict of interest statement*. None declared.
